# Healthy Eating and Active Living: Rural-Based Working Men’s Perspectives

**DOI:** 10.1177/1557988315619372

**Published:** 2015-12-14

**Authors:** John L. Oliffe, Joan L. Bottorff, Paul Sharp, Cristina M. Caperchione, Steven T. Johnson, Theresa Healy, Sonia Lamont, Margaret Jones-Bricker, Kerensa Medhurst, Sally Errey

**Affiliations:** 1 University of British Columbia, Vancouver, British Columbia, Canada; 2 University of British Columbia, Kelowna, British Columbia, Canada; 3 Athabasca University, Alberta, Canada; 4 Northern Health, Prince George, British Columbia, Canada; 5 British Columbia Cancer Agency, Vancouver, British Columbia, Canada; 6 Canadian Cancer Society, Prince George, British Columbia, Canada; 7 Australian Catholic Universality, Victoria, Australia

**Keywords:** men’s health, masculinity, physical activity, healthy eating, health promotion

## Abstract

There is a pressing need for health promotion programs focused on increasing healthy eating and active living among “unreached” rural-based men. The purpose of the current study was to describe rural-based working men’s views about health to distil acceptable workplace approaches to promoting men’s healthy lifestyles. Two focus group interviews included 21 men who worked and lived in northern British Columbia, Canada. Interviews were approximately 2 hours in duration; data were analyzed using thematic analysis. Themes inductively derived included (a) food as quick filling fuels, (b) work strength and recreational exercise, and (c) (re)working masculine health norms. Participants positioned foods as quick filling fuels both at work and home as reflecting time constraints and the need to bolster energy levels. In the theme work strength and recreational exercise, men highlighted the physical labor demands pointing to the need to be resilient in overcoming the subarctic climate and/or work fatigue in order to fit in exercise. In the context of workplace health promotion programs for men, participants advised how clear messaging and linkages between health and work performance and productivity and cultivating friendly competition among male employees were central to reworking, as well as working, with established masculine health norms. Overall, the study findings indicate that the workplace can be an important means to reaching men in rural communities and promoting healthy eating and active living. That said, the development of workplace programs should be guided by strength-based masculine virtues and values that proactively embrace work and family life.

## Introduction

Engaging in healthy lifestyle behaviors including healthy eating and physical activity has been directly linked to reduced risk of developing chronic illnesses and cancer (Khaw et al., 2008; Lee et al., 2012; World Health Organization, 2004); however, many men continue to live unhealthy lifestyles (Bauman et al., 2009; Northern Health, 2010; Shay et al., 2012; World Cancer Research Fund International, 2007). For example, few men engage in the recommended levels of physical activity (Kohl et al., 2012; World Health Organization, 2010; 150 minutes or more of moderate intensity physical activity per week), and many men overuse alcohol (Kuntsche, Knibbe, & Gmel, 2009) and have diets that are calorically dense and low in fruits and vegetables (Gough, 2007; Wardle et al., 2004). Men can be reticent to seek advice from health care professionals, attend lifestyle education sessions, and/or independently seek out health promotion information (Caperchione et al., 2012; Deeks, Lombard, Michelmore, & Teede, 2009; Yousaf, Grunfeld, & Hunter, 2014). These common male practices have also been cited as deeply implicated in reports that men, compared with women, have a shorter life expectancy and experience higher morbidity rates associated with chronic disease (Pinkhasov et al., 2010; Salomon et al., 2012).

Ineffectual efforts to engage men in physical activity and healthy eating programs have been linked to failures to integrate gender into the design and implementation of health promotion initiatives (Bottorff et al., 2015; George et al., 2012; Gray et al., 2009; Morgan et al., 2011). The influence of masculinity has featured in commentaries and discourse analyses about men’s inactivity and poor diets (Courtenay, 2000; Gough, 2007). There is however little research describing remedies for engaging “unreached” male populations including men who live and work in rural communities (Carroll, Kirwan, & Lambe, 2014). From a social constructionist gender theory viewpoint, while a plurality of masculine performances have been anchored to men’s health and illness, Cormie et al. (2015) have argued that to acknowledge and operationalize specific masculine virtues and values including self-reliance, authenticity, and provider and protector roles can advance the health of men and their families. Place also intersects with masculinity to have a significant impact on specific men’s health practices. For example, men living in rural and regional areas in Canada are more likely to have low levels of education, be employed in hazardous jobs, and exhibit high health risk behaviors associated with chronic disease (Canadian Institute for Health Information, 2006). The purpose of the current study was to describe rural-based working men’s views about health to distil acceptable workplace approaches to promoting men’s healthy lifestyles.

## Method

### Study Site

Prince George is located in the north of British Columbia, and with a population of 71,974 (Statistics Canada, 2011), it is, by definition, a city with considerable infrastructure, including a public hospital and a university. Though Prince George is a city in this regard, it is also a rural place with regard to aspects of local culture, northern location, and the availability of employment in natural resource sectors. Indeed, resource extraction sectors shape the region, and more men are employed in trades, transport, and equipment operator and related occupations than any other single sector (33% of all men in the labor force; Statistics Canada, 2007). The area is home to pulp and paper mills, lumber sawmills, plywood manufacturing, an oil refinery, and chemical plants. Sizable oil and mineral deposits in the area have also given rise to additional resource industries and employment opportunities.

What constitutes “rural” is widely contested in geography and Prince George, as a conventionally defined city with a rural character, is illustrative of the instability of a binary distinction between urban and rural, or of any singular rural type (Campbell, Bell, & Finney, 2006; Cloke, 2006; Woods, 2009). As such, adopted was the self-ascribed rural classification of Prince George put forth by the study participants. In terms of illness, the prevalence rates of all cancers, cardiovascular disease, hypertension, asthma, and chronic obstructive pulmonary disorders in northern British Columbia are high compared with other health regions (Provincial Health Services Authority, 2010), and higher in men than women (Kushi et al., 2012).

### Recruitment and Sample

Following University ethics approval, men (18 years and older) who lived and worked in northern British Columbia were recruited using printed posters, online media (e.g., Kijiji, Castanet, Facebook), and targeted telephone calls to potential participants. The advertisements invited men to take part in focus group interviews to share their perspectives about healthy eating and active lifestyles in the context of workplace health promotion. A total of 41 men contacted the project coordinator, and further details about the study, including how the information shared would be used to inform the design of workplace health promotion programs for men, were provided to potential participants. Two focus group interviews were conducted with a total of 21 participants. The men ranged in age from 23 to 73 years (*M* = 46; *SD* = 11.5) and worked in a variety of jobs and industries connected to Prince George’s resource sectors (e.g., millworker, painter, short-haul driver). The majority of participants (85%) were overweight to obese. Data regarding the men’s dietary options and opportunities to engage in physical activity at work were also collected along with self-reported body mass index details (see Table 1).

**Table 1.** Description of Sample and Workplace Characteristics (*N* = 21).

Characteristic	*n* (%)
Age (years)	
20-29	2 (10)
30-39	2 (10)
40-49	9 (42)
50-59	5 (24)
60+	3 (14)
Education	
Less than high school	2
Completed high school	8
Trades certification	8
Completed college or university	3
Ethnicity	
Caucasian	17
Aboriginal	2
European	1
Other	1
Occupation	
Trades, transport, and equipment operators and related occupations	15
Sales and service occupations	4
Business, finance, and administration occupation	2
Availability of food in workplace	
No food available, bring my own	7
Snack food is available, no meals	9
Meals available for purchase, but few healthy options	1
Meals available for purchase, including healthy options	2
Meals are provided, but few are healthy	1
Healthy meals provided	1
Availability of exercise facilities in workplace	
Yes	7
No	14
Percentage of time spent sitting at work	
<10	6
10-29	0
30-49	3
50-69	4
>70	8
Percentage of time spent walking at work	
<10	7
10-29	10
30-49	2
50-69	2
>70	0
Participation in recreational sporting activities	
Yes	14
No	7
Body mass index (kg/m^2^)	
Normal weight (18.5-24.9)	3
Overweight (25.0-29.9)	12
Obesity Class I (30.0-34.9)	5
Obesity Class II (35.0-39.9)	1
Obesity Class III (>40)	0

### Data Collection

The two focus group interviews, each lasting approximately 2 hours were conducted in May 2014. Using a semistructured approach, questions and activities were integrated to engage participants and promote discussion. For example, opportunities for storytelling and small group discussions were used to explore specific topics and details in depth. Three of the authors facilitated the focus group sessions (SJ, TH, PS) asking open-ended questions including “How does living and working in the north influence the way men take care of their health?” and “What kinds of things do northern men do or like to do that help them stay healthy?” One facilitator took a lead in easing the conversations, while two others asked probing questions and assisted with the small breakout group discussions. The interviews were digitally recorded and a fourth team member (KM) kept a speakers log to connect participant’s quotes in the transcribed record of the interviews. The small group breakout sessions were also facilitated, digitally recorded, and transcribed with researcher observations added into the transcripts. The breakout groups were included to ensure participants were active in the process, and to discuss the use of the pedometers given to the men at the beginning of the session, which they later checked to look at how many steps they had accumulated as a means to discussing the potential for pedometers to motivate men’s physical activity. Men’s health promotion sample materials were posted on the focus group meeting room walls, and in pairs or groups of three, men circulated stopping at each poster to discuss the key messages and evaluate the appeal from their perspective. In addition, participants provided advice to guide the design and implementation of men’s workplace health promotion programs in male-dominated worksites. Observing the focus group and small group breakout sessions revealed the men as quick to engage one another in voicing their opinions, and the permission of participants to discuss health-related issues and potential remedies was central to promoting the men’s dialogue. All participants provided written informed consent and received a $50 honorarium to acknowledge their contribution to the study and offset travel costs.

### Data Analysis

The focus group sessions were transcribed verbatim and checked for accuracy. Potentially identifying information (e.g., specific workplaces, colleagues, and managers) was removed to aid anonymity. Participants were also assigned an identification number in the speaker’s log which in turn was linked to participant’s quotes in the transcribed record. The interview data were read independently by the team members and through discussion a coding schedule was developed. Codes included masculinity, northern influences, and man messaging, to which data segments were allocated using NVivo 10 (QSR International®). Each of the codes and the corresponding data were subsequently reviewed by the authors to inductively derive understandings and preliminary themes, within and across the data. Three themes were developed: (a) food as quick filling fuels, (b) work strength and recreational exercise, and (c) (re)working masculine health norms. These themes are discussed and summarized in the results section, and the findings are theorized using masculinities (Connell, 2005) and place (Massey, 1994) frameworks.

## Findings

The men positioned their ability to take up a healthy lifestyle in the context of living and working in northern British Columbian communities. As such, their commentaries were punctuated with references to the influence of the subarctic climate, the long distances between communities and work and home, and the uninhabited wilderness that could be isolating amid providing unique recreational opportunities. In addition, work, family life, and “breadwinner” responsibilities featured in the men’s conversations. Participants prided themselves on their ability to work in, and overcome harsh conditions, and awareness of healthy eating and active living were entwined with workplace and family responsibilities.

### Food as Quick, Filling, Fuels

For the most part, from the researcher’s perspectives, participants were aware of what might constitute a healthy diet. However, men’s appetite and challenges around time management converged with locale specific food supply and access issues to influence participants’ positioning of food as quick, filling fuels. Within this context, paid work was central and food was a means to an end in garnering the energy for “getting the job done.” For example, men working in transport and resource extraction industries detailed their demanding schedules, highlighting the isolation of working far from home for extended periods of time. Within these spaces, the food choices were limited to what was easily accessible. Participants emphasized how quick food enabled them to keep working and balance the demands of family life. A 47-year-old highway truck driver explained:

You get into that lifestyle, the quick food, because you haven’t got the time when you’re knocking off 100 miles away from home. You’re looking to try and maximize as much time as you can with your family so a lot of times it’s the quickest thing you can do, and it’s not always the healthiest.

Evident here was how the demands of working remotely could extend to involve a significant commute, which in turn reduced men’s time with family. In addition, many men explained that because of their work schedules, family time was rarely organized around cooking and/or sharing a home cooked family meal together. Instead, many participants also resorted to quick and easy food options at home. The work and domestic spheres merged somewhat in this respect, wherein many men were chronically time poor, juggling the priority and demands of work with family life in ways that routinely resulted in taking up quick food options. Embedded in these quick food practices were northern norms, as a 43-year-old truck driver said, “The way my friends eat is kind of the way we eat.”

Also, central to the use of quick foods was their filling capacity. In contrasting and rationalizing the need for bulk and energy sources, participants pointed to the shortcomings of healthy options for quelling their appetite in sustained ways. As a 45-year-old welder explained:

You can eat healthy, the fruit and vegetables, but in 2 hours you’re hungry. You can’t just stop work and eat whenever right? And that’s the problem. You have 15 minutes to eat. A guy wants to eat, he wants food. You want to eat something that’s gonna work, right? A couple carrots and some lemons and stuff like that—in 15 minutes, in a half hour you’re hungry again and that’s the thing. It’s healthier, but you gotta eat more consistently and more times during the day in order to actually benefit from it, right. And that’s a big hurdle. I think that’s a hurdle for more guys.

This man’s quote revealed issues around the workday affording him limited time to eat, perceptions round the inability to feel sated when consuming fruits and vegetables and his need to fill up quickly and continue to work. Similar challenges around scheduling meal breaks were shared by a 46-year-old engineering technician who explained, “It was hard for me to eat steady because I was getting my hands dirty so I had to eat at certain times.” Though some participants, including the truck drivers, could carry food supplies with them and had the opportunity to “graze” the remoteness and transient nature of their work revealed how the lack of infrastructure could reduce their access to healthy meal and food options. As a 47-year-old truck driver stated:

The problem up north here is we’ve got such great distances between centers, small population, everybody’s after the quick dollar. Nobody, unfortunately, due to economics and circumstances, nobody keeps stock anymore. So, everything moves day to day. But, all that stuff moves usually at night. Well, nothing’s open. So again, the drivers, a lot of them don’t have options. There’s nothing there.

Countering a reliance on vendors, a few participants asserted that hunting and fishing in the north could adequately provide fresh and healthy food options. As a 52-year-old saw filer explained, “You go and you kill something and you eat it. That’s kind of the way we’re living [here].” While catching wild game and fish was espoused as promoting healthy eating, some participants recognized the limitations, including one 73-year-old sales and security associate who countered:

I don’t think the north is conducive to eating better. . . . People eat moose, pig, beef, and whatever, but there’s a lot of things on the other side that we’re maybe not getting enough of. . . . In the winter time, it’s mashed potatoes and meat, and vegetables are really not available.

Complicating matters, hunting and fishing require men having the resources, skill, and time to pursue these activities. Revealed here were diverse alignments to northern masculine ideals wherein participants could embrace or distance themselves from the benefits of northern foods and/or the utility of men’s resourcefulness in securing those types of food options.

### Work Strength and Recreational Exercise

Participants, especially those working physically demanding jobs contrasted the strength required to labor, as well as the physical conditioning drawn from that work, with recreational and/or family-related exercise. A 47-year-old truck driver suggested that in his workday, “You’re going to get a workout whether you like it or not.” He explained that he regularly moved a “3000 or 4000 lb converter around by hand” in asserting that because he “worked hard” having to exercise “on top of that” was neither feasible nor necessary. Evident in this man’s interview, and other like-minded participants, was a recursive relationship wherein the strength to labor, and strength and conditioning derived from that physical work, diminished the need for recreational or incidental physical activity. The focus of most participants was also on physical strength rather than aerobic capacity and exercise fitness. A 45-year-old welder/mechanic countered the traditional conventions by which fitness was evaluated:

They don’t take into account a guy that works physically all day and he gains mass from it. If I was 120 pounds, there’s no way I could fling steel around.

Herein body mass and particular body types were understood as central to the physicality required to do specific work, and these embodied ideals were differentiated and distanced from lean bodies and/or aerobic capacity. The men’s work also resulted in some fatigue, as a 42-year-old firefighter suggested, “You get home, put your feet up and have a bag of chips.”

Indeed, outside of the direct context of paid work a range of locale specific factors emerged as barriers and facilitators for participants engaging in meaningful physical activity. For example, while living in the north was synonymous with the outdoors, the cold harsh winters could deter efforts to be physically active. As a 44-year-old firefighter explained:

I’m not big “on going to the gym guy.” I like to be outdoors. But when the [winter] weather is the shits for 2 to 3 weeks at a time, I just kind of huddle up indoors.

Coupled with climate challenges, some men explained that they put their children’s physical activities and sports commitments ahead of their own exercise. The time and costs associated with supporting these family activities in turn was put forth as eroding the capacity for some participants to have their own recreational exercise regimen.

Overshadowing the barriers were participant commentaries suggesting the climate challenges were exaggerated. Offered instead, were assertions that the region had unique opportunities to be active and taking advantage of that was reliant on people “being flexible” and having the “right attitude.” As a 46-year-old engineering technician explained, “I’m trying to make the most of it and I see there’s a lot more here than I give credit for.” Similarly, a 53-year-old technician affirmed the north as affording him and his family a wide range of exercise-related experiences:

Three winters ago as a family we came here because we were all energetic and like the recreational stuff outside. There are two seasons [in Prince George], summer life and winter life. I think it’s easy to fulfill, being active outside [in summer], but winter becomes a bit more of a challenge. So, we bought five sets of snowshoes three Christmas’ ago . . . and we have a recreational cabin. We take them up there, snowshoe up there when the conditions are right.

Contrasts between the north and urban centers were also made in pointing to the cheaper golfing and gym membership options in Prince George. These narratives collectively defended and focused on what Prince George and life in northern communities had to offer, amid pointing to the resilience and innovation to fully muster those opportunities. Similarly, counter claims were made by many men, about the centrality of family for being active. A 53-year-old technician explained that his wife valued regular physical activity and would “kick [him] off the couch and say ‘hey, let’s go’” if he had not been to the gym in several days, while a 47-year-old sheriff pointed to the positive influence of his children:

You need your family. You can be tired and they say, “Come on dad, let’s go.” Let’s go for a walk, let’s go to the gym. That kind of helps, that kind of motivates you.

Men also highlighted the value of being a good role model in being healthy and able to “keep up” with their children. A 47-year-old truck driver boasted, “I told my girls I want to be the 80-year-old that is ripped in the gym and jogging 10 kilometers.” Traded on here, were long-standing masculine norms where men do self-health for others—most often, family.

Overall, most participants revealed their appreciation for all that the north had to offer in terms of physical activity options while conceding that it took some effort to consistently engage with recreational physical activity. The triage of work strength, above recreational physical activity was also ever present in the men’s accounts, wherein work demanded and got much of the participant’s energy.

### (Re)Working Masculine Health Norms

Most men understood that being healthy would aid their ability to secure and sustain paid work, and within this context healthy eating and physical activity were seen as key to being fit to work. Participants also agreed, at least in principle, that the workplace was a viable environment for advancing men’s health. However, the men were clear that the ways in which workplace health programs were presented were critically important to men’s willingness to engage such initiatives. First and foremost, the foundation for success resided in convincing men that there were benefits to participating, as a 43-year-old truck driver warned:

If they [men] don’t want to do it, they’re not going to do it. Doesn’t matter what you throw at them, if they can’t see it’s for their benefit and they can’t see that it is going to make their life better, they’re not going to want to do it. It’s not until you can get them to want to do it, you might as well bang your head on the wall.

This assertion drew strong agreement from fellow focus group participants, and collectively expressed was the need for program designers to understand that men would critically judge the usefulness and relevance of workplace health initiatives in deciding their involvement. Embedded also was the participants’ familiarity with masculine cultures characterized by men’s autonomy and decisiveness. Related to this, participants explained that one of the biggest challenges involved changing specific masculine norms within male-dominated workplaces. For example, healthy eating within the workplace, where filling and fuel food choices were normed, risked significant ridicule from male peers. A 61-year-old pulp mill worker explained how straying outside the workplace cafeteria meat and potato option and eating salad and chicken drew the following from his male coworkers, “What the hell are you eating? That’s not good for you.” Similarly, a 52-year-old saw filer lamented the policing that occurred around him bringing his lunch and eating *different* foods:

You know I go to work with my lunch and the hardest part for me of trying to eat the way I think is healthy, it’s other guys’ attitudes. I get ridiculed. They say “what the hell are you eating?” that kind of thing.

Herein dominant ideals about men eating “manly” foods were reaffirmed, and eating healthy was “othered” as unmanly or worse still, feminine (e.g., eating salad). In this context, the risks associated with transgressing work cultures might outweigh the potential benefits of eating healthy, a tension also threatening the overall feasibility of men’s workplace health promotion programs.

In terms of breaking with such restrictive masculine norms, participants offered a range of important strategies. For example, the ridiculing cultures referred to in the aforementioned examples were also understood and explained by some participants as a form of camaraderie, and with the right “friendly competition” health promotion programs, these dynamics could be capitalized on to support men to make healthy choices. A 47-year-old truck driver related a story about healthy food choices in this regard:

Jokingly, we start teasing each other about how much coffee we were drinking and okay let’s replace it with a bottle of water a night. Fruit, well how much fruit did you eat today? How many veggies did you eat today?

These friendly challenges imbued with humor provided opportunities for some men to compare their performance and outcomes, as a 41-year-old corrections officer suggested, “So-and-so does something, we all hear about it and then from there it’s all one-upmanship.” Similarly, the influence of the group’s actions was understood as strong drivers for changing and challenging norms around exercise. A 34-year-old warehouse and courier manager suggested, “When everyone else is going out you can’t be the only one that’s sitting.” These poignant examples revealed how some masculine norms were reworked to promote rather than restrict healthy eating and physical activity in the workplace. Indeed, an individual or small group of men within the workplace could champion such change. For example, observing weight loss in men eating healthy was cited as a strong motivator, and the 73-year-old sales and security associate explained how this *proof* could shift norms, “If someone is eating healthy then you tend to eat what they’re eating.” Senior management were also understood as potent change agents, as a 46-year-old engineering technician explained:

A couple of guys were talking, and . . . they said, “Well why don’t we get like sandwiches or veggies.” That’s what I’m actually going to mention to the boss next time because . . . that’s one of my jobs is to find out what the guys want and to mention it whether it’s a coffee maker or healthy food options. So it’s kind of interesting that guys are interested in healthy food.

Participants warned that workplace program content and its marketing messages needed to be “simple” with “hard stats,” visual information (“because guys are visual”) and provide “tangible” ways of monitoring progress. A 46-year-old engineering technician explained:

It needs to be simple, so men will understand it. And they need to know quantitatively what is healthy. You know protein, so how much a day, what is a protein, what is a serving of protein. . . . Show what a serving size is [so] you don’t have to wonder, you don’t have to worry about calories. It’s just a fistful, one baseball. That’s geared to men.

This participant, along with many others confirmed the need to focus on what was eaten in enabling men to choose and monitor their intake, as distinct from a focus on dieting which was implicit to excluding some foods and counting calories. There was also strong support for positively framing messages to promote healthy eating. A 42-year-old firefighter argued that cause–effect relationships explicitly linking health to benefits round work performance and productivity drove men toward certain foods

You start eating this kind of food, this is what’s going to happen, you’re going to add this to your life. You’re going to add momentum and strength and you’re going to be able to work longer you’re going to be able to decrease your injuries and so on.

In summary, working and reworking some masculine health norms were central to participants’ advice for engaging men in workplace health promotion programs. In terms of convincing employees, many participants argued that employers should incentivize men’s participation in and beyond workplace health promotion programs. As a 45-year-old welder/mechanic pointed out, supporting men to take up recreational exercise was also key to having healthy employees:

I think the employers need to be educated that the healthier . . . [we are] at work, the more beneficial we are to them in the long run. Exercise and stuff . . . if they’re going to expand on helping guys with gym dues or whatever, you know, even partial, it will make a huge difference.

## Discussion and Conclusion

The current study findings provide important contexts for thoughtfully considering and perhaps reconsidering some of what is claimed as predominant male health practices round diet and physical activity. Food and men’s health research, for example, has highlighted how dominant masculine discourses can direct men’s food choices (Gough, 2007), the influence of culture and immigration on food and alcohol consumption (Oliffe et al., 2010), and connections between male obesity and the marketing of better-for-you foods and beverages (White, Oliffe, & Bottorff, 2014). Adding to these insights, the current study findings reveal how northern masculinities can shape, as well as be shaped by dominant ideals about what men eat. Herein much is on offer in terms of potential remedies toward promoting healthy eating among men at work—because the strategic actions of a few men can model and permit and affirm others to collectively rework masculine ideals about men eating healthy. Increased work performance and productivity as by-products of eating healthy also play strongly to masculine workman ideals, and building on this highlighting such cause–effect relationships can be a potent change agent. The potential for healthy eating to also flow from the workplace to the domestic sphere might be realized over time.

In the context of physical activity, affirmed are place specific findings from Gavarkovs, Burke, and Petrella (2015) who highlighted fatigue, time constraints, and “I think I get exercise at work” as the three most cited barriers to regular physical activity among rural Canadian men. Building on this, the current study findings highlighted divisions between work and home, and strength and aerobic fitness in ways that clearly differentiated men’s paid labor from recreational physical activity. In terms of potential remedies for promoting physical activity at work Wong, Gilson, van Uffelen, and Brown (2012) suggest that few targeted men’s programs have been designed or rigorously evaluated. Similarly, a review by Bottorff et al. (2015) reported that only 12 programs including promotion of physical activity explicitly integrated gender-related influences in male-specific programs that recognized men’s interests and preferences. The findings drawn from the current study confirm that men triage where and how they expend their energy with paid work consistently trumping recreational physical activity. With this in mind, benefits might be garnered by building physical activity into the workdays of rural men to bolster their productivity, rather than positioning it as something that should be reliant on recreation time. Accommodating well established gender norms—such as “friendly” competition and catering to methods and modes of delivery that recognize and exploit gender differences (Caperchione et al., 2012) promise improvements in uptake and success for workplace-based models. Additionally, paying attention to place, featuring the workplace, or context of men’s place in the world is important: In this case, northern men wanted to see northern men and northern work sites present in visual images and other materials. A poignant example of this occurred in the focus group interviews where participants quickly critiqued the use of an image featuring a foreign truck in the promotional materials, pointing out that it was not a truck that would be used in the north. This really spoke to how the program look, feel, and language needed to fit the group so that disconnects and dismissal did not occur at first glance by the potential end users.

The potential benefits of men’s workplace health promotion programs, especially in male dominated work environments are encouraging. For example, men’s mental health can improve with programs focused on physical health, and this is vitally important given the rise in workers compensation payouts related to mental illness (Oliffe & Han, 2014). That said, high unemployment, long-term economic uncertainty, and ever vigilant attention to the “bottom-line” company profits has rendered many male workers, especially those employed in resource extraction sectors, somewhat disposable in the eyes of some employers. In this regard, longitudinal studies reporting relationships between sustained targeted men’s health promotion programs and employee absenteeism, retention and recruitment might influence employers to invest in targeted health promotion initiatives.

In terms of limitations, the current study is reliant on a small participant cohort and the findings are contextually tied to specific locales and workplaces. In this regard, the narrow focus limits what might be claimed to likely prevail in other locations. The cross-sectional nature of the study design also limits what can be said about potential changes across time. These limitations however provide some direction for future research which might usefully include larger longitudinal and mixed methods studies to distil with greater certainty the similarities and differences for men’s health practices in rural locales.

In conclusion, the findings from the current study confirm the need for gender sensitive men’s workplace health promotion programs that are attuned to place specific rural cultures and norms. This was especially evident in how men’s health practices responded to and reflected Prince George ideals as well as specific workplace and gender norms. In turn, the effectiveness of potential remedies is deeply reliant on knowing, engaging, and working with, as well as reworking some masculine ideals about how men engage in healthy lifestyles in specific locales. In this regard, the participant’s engagement, curiosity, and willingness to tackle questions and ideas within the focus groups provided important insights about useful strategies for engaging and supporting men.
